# Increased prevalence of minor physical anomalies in patients with epilepsy

**DOI:** 10.1038/s41598-022-17853-1

**Published:** 2022-08-12

**Authors:** Dalma Tényi, Tamás Tényi, Györgyi Csábi, Sára Jeges, Beáta Bóné, Katalin Lőrincz, Norbert Kovács, József Janszky

**Affiliations:** 1 Department of Neurology, University of Pécs, Rét u 2, Pécs, 7623 Hungary; 2 Department of Psychiatry and Psychotherapy, University of Pécs, Pécs, Hungary; 3 Department of Pediatrics, University of Pécs, Pécs, Hungary; 4 Institute of Nursing and Patients Care, Faculty of Health Sciences, University of Pécs, Pécs, Hungary

**Keywords:** Epilepsy, Neurodevelopmental disorders

## Abstract

Our aim was to investigate the rate and topological profile of minor physical anomalies (MPAs) in adult patients with epilepsy with the use of the Méhes Scale, a comprehensive modern scale of dysmorphology. Consecutive epilepsy patients admitted for outpatient evaluation were included. Patients with comorbidities of neurodevelopmental origin (such as autism, severe intellectual disability, attention deficit hyperactivity disorder, schizophrenia, tic disorder, Tourette syndrome, bipolar disorder, specific learning disorder and specific language impairment) were excluded. All participants underwent physical examination with the use of the Méhes Scale for evaluation of MPAs, including 57 minor signs. The frequency and topological profile of MPAs were correlated to clinical patient data using Kruskal–Wallis, chi2 tests and logistic regression model. 235 patients were included, according to the following subgroups: acquired epilepsy (non-genetic, non-developmental etiology) [N = 63], temporal lobe epilepsy with hippocampal sclerosis (TLE with HS) [N = 27], epilepsy with cortical dysgenesis etiology [N = 29], cryptogenic epilepsy [N = 69] and idiopathic generalized epilepsy (IGE) [N = 47]. As controls, 30 healthy adults were recruited. The frequency of MPAs were significantly affected by the type of epilepsy [H(6) = 90.17; p < 0.001]. Pairwise comparisons showed that all patient groups except for acquired epilepsy were associated with increased frequency of MPAs (p < 0.001 in all cases). Furrowed tongue and high arched palate were more common compared to controls in all epilepsy subgroup except for TLE (p < 0.001 or p = 0.001 in all cases). A positive association was detected between the occurrence of MPAs and antiepileptic drug therapy resistance [Exp(B) = 4.19; CI 95% 1.37–12.80; p = 0.012]. MPAs are more common in patients with epilepsy, which corroborates the emerging concept of epilepsy as a neurodevelopmental disorder. Assessment of these signs may contribute to the clarification of the underlying etiology. Moreover, as increased frequency of MPAs may indicate pharmacoresistance, the identification of patients with high number of MPAs could allow evaluation for non-pharmacological treatment in time.

## Introduction

Epilepsy is a large, heterogenic group of chronic neurological conditions characterized by spontaneously recurring seizures and comprises various syndromes that differ in etiology. In many epilepsy syndromes, previously assumed to be caused by one specific anomaly (e.g. channelopathy in juvenile myoclonic epilepsy or hippocampal sclerosis in temporal lobe epilepsy), it has been recognized that the genetically and environmentally determined prenatal proliferation, migration and organization of neuronal and glial cells and the postnatal maturation of neuronal networks also contribute to the epileptogenesis^1–4^. The recent discovery of these developmental factors’ additional role in pathogenesis gave rise to the concept of epilepsy as a neurodevelopmental disorder^1^.

Minor physical anomalies (MPAs) are subtle, clinically and cosmetically insignificant errors of morphogenesis but may be of major informational value for diagnostic, prognostic and epidemiological purposes^5,6^. Since the surface ectoderm (which later forms the skin) and the neuroectoderm differentiate from the same ectodermal tissue early in gestation, MPAs may serve as sensitive external markers of abnormal neurodevelopment, such as in—among others—autism, attention deficit hyperactivity disorder or schizophrenia^5–9^. They are considered to develop during the first and/or early second trimester and since they persist into adult life, they can be detected on physical examination at any age from neonates to the elderly.

As we^10,11^ and others^12^ have pointed out previously, the contradictions and differences among studies on MPAs in different neurodevelopmental disorders can be—at least partly—ascribed to the problems with the use of the Waldrop-scale for the detection of these signs, which lists only 18 MPAs and does not differentiate between minor malformations (MM) which form during organogenesis and phenogenetic variants (PV) which arise after organogenesis^6,11,13–15^. Addressing these concerns, a comprehensive new scale was developed by Méhes, listing 57 MPA’s and clearly defining them as either MM or PV. With the use of the Méhes Scale we have detected increased frequency of MPAs in patients with neurodevelopmental disorders including schizophrenia^10,16,17^, Tourette syndrome^18^, major depression^19,20^, autism^21^, bipolar affective disorder^22,23^, fetal alcohol syndrome^6^, intellectual disability^6^, cerebral palsy^6^ and alcohol dependence^10^.

In the first part of the twentieth century, a few studies reported on increased frequency of MPAs in epileptic patients^24–26^, however, these were not carried out with the use of scales developed based on evidence of modern dysmorphology. In a pilot study on children with epilepsy, analyzing 24 subjects and 24 controls, we detected an increased frequency of MPAs in patients with cryptogenic childhood epilepsy^27^. The aim of the present study was to investigate the rate and topological profile of MPAs in adult patients with epilepsy with the use of a comprehensive modern scale of dysmorphology for the first time. Furthermore, the large sample size allowed us to assess these signs also in terms of etiology, symptomatology and anamnestic factors.

## Methods

Consecutive patients with diagnosed epilepsy were included who were admitted for outpatient evaluation or consultation to the Department of Neurology, Clinical Center of University of Pécs between January 2016 and August 2018. Patients with comorbidities of neurodevelopmental origin (autism, severe intellectual disability, attention deficit hyperactivity disorder, schizophrenia, tic disorder, Tourette syndrome, bipolar disorder, specific learning disorder and specific language impairment) were excluded. As controls, healthy adults were recruited from the ward’s health care providers. All participants underwent physical examination with the use of the Méhes Scale for evaluation of MPAs, including 57 minor signs, clearly differentiated either as MMs or PVs, as presented in Table 1. All items from the Waldrop scale were included in our list except for head circumference and longer third toe. The physical examination was performed by an investigator of our research team (D.T.) with years of experience in clinical dysmorphology, who has previously participated in more studies on MPAs with measured strong interrater reliability^17,27^. The examination was performed qualitatively (present or absent) without scores being used, although—where it was possible—measurements were taken with calipers and tape to improve objectivity. The standards and techniques of measurement of MPAs were carried out in accordance with the instructions by Feingold and Bossert^28^ and Méhes^6^.

**Table 1:** The Méhes Scale.

Minor malformations	Phenogenetic variants
Preauricular tag	Small mandible
Preauricular pit	Confluent eyebrows
Lip pit	Short palpebral fissures
Bifid uvula	Mongoloid slant
Supernumerary nipples	Antimongoloid slant
Partial syndactily of toes 2–3	Inner epicanthic folds
Pigmented naevi	Hypertelorism
Café-au-lait spots	Asymmetrical size of ears
Haemangioma	Protruding auricle
Sacral haemangioma	Low set of ears
Prominent occiput	Abnormal philtrum
Prominent forehead	Large or small oral opening
Flat forehead	High arched palate
Flat occiput	Large tongue
Primitive shape of ears	Short sternum
Cup ears	Wide-set nipples
Earlobe crease	Acromial dimples
Simian crease	Deep sacral dimple
Sydney line	Unusual length of fingers
Single flexion crease on the 5th finger	Clinodactily
Sole crease	Hallucal abnormality
Prominent heel	Wide distance between 1st and 2nd toes
Double posterior hear whorl	Nail hypoplasia
Multiple buccal frenula	Dimple on the tuberositas tibiae
Furrowed tongue Brushfield spots Fine electric hair Tongue with smooth and rough spots Frontal upwap Lack of earlobe Double anthelix	Dimple on the elbow

The frequency and topological profile of MPAs were correlated to clinical patient data: type of epilepsy, age at onset of epilepsy, etiology, response to antiepileptic drug therapy and family history of neurodevelopmental disorders. MRI sequences were chosen and analyzed according to epilepsy protocol. The diagnosis of the patients was established in accordance with the International League Against Epilepsy (ILAE) Classification of the Epilepsies^29^. We defined drug resistant epilepsy based on the 2010 Consensus proposed by the task Force of the ILAE Commission on Therapeutic Strategies^30^.

The study performed was part of a study series on minor physical anomalies approved by the Ethical Committee of the University of Pécs. Informed consent was obtained from all patients. We confirm that we have read the Journal’s position on issues involved in ethical publication and affirm that this report is in accordance with relevant institutional guidelines.

For general sample characteristics, descriptive statistical methods were applied. For group comparisons, Mann–Whitney, Kruskal–Wallis and Fisher exact tests with Bonferroni correction were used. To determine the association between the presence of minor physical anomalies and therapy resistance, binary logistic regression model was applied. Multinomial logistic regression model was designed to compare epilepsy subgroups in terms of MPAs. Statistical analysis was carried out with the use of IBM SPSS Statistics 22.0.

## Results

Based on the inclusion criteria, 235 patients were included in our study, according to the following subgroups: acquired epilepsy, temporal lobe epilepsy with hippocampal sclerosis (TLE with HS), epilepsy with cortical dysgenesis etiology, cryptogenic epilepsy and idiopathic generalized epilepsy (IGE). Acquired epilepsy, that is a group of epilepsy patients with non-genetic, non-developmental etiology (further on will be referred as “acquired epilepsy”) consisted of patients with post-inflammatory, posttraumatic, poststroke, postoperative epilepsy and epilepsy associated with brain tumor or birth injury. IGE subgroup consisted of patients with juvenile myoclonic epilepsy (JME), childhood absence epilepsy (CAE), juvenile absence epilepsy (JAE) and epilepsy with generalized tonic–clonic seizures (E-GTCS). As controls, 30 healthy adults were recruited. The mean age of patients was 48.6 ± 14.6 years. The mean age of controls was 44.2 ± 13.17 years, 17 of them were male. Table 2 displays the total count of MPAs of subjects across the different patient groups and controls. Table 3 displays the general data of our cohort in terms of patient characteristics, anamnestic data and the frequency of MPAs.

**Table 2:** The total number of minor physical anomalies in each patient in the different epilepsy subgroups.

	Number of minor physical anomalies						
	0	1	2	3	4	5	6
Control	14 (47%)	12 (40%)	4 (13%)	0	0	0	0
Acquaired epilepsy	12 (19%)	20 (32%)	18 (28%)	9 (14%)	1 (2%)	3 (5%)	0
TLE with HS	1 (4%)	4 (15%)	9 (33%)	7 (26%)	4 (15%)	2 (7%)	0
Epilepsy with cortical dysgenesis	0	3 (10%)	6 (21%)	8 (28%)	6 (21%)	4 (14%)	2 (7%)
Criptogenic epilepsy	6 (7%)	15 (22%)	17 (25%)	18 (26%)	10 (14%)	2 (4%)	1 (2%)
**IGE (altogether)**	2 (8%)	2 (8%)	4 (15%)	8 (30%)	8 (31%)	2 (8%)	0
JME	0	1 (5%)	1 (5%)	5 (24%)	6 (28%)	7 (33%)	1 (5%)
CAE	0	0	0	1 (100%)	0	0	0
JAE	0	1 (8%)	2 (17%)	4 (33%)	5 (42%)	0	0
E-GTCS	2 (15%)	1 (8%)	2 (15%)	3 (23%)	3 (23%)	2 (15%)	0

*CAE* childhood absence epilepsy, *E-GTCS* epilepsy with generalized tonic–clonic seizures, *HS* hippocampal sclerosis, *IGE* idiopathic generalized epilepsy, *JAE* juvenile absence epilepsy, *JME* juvenile myoclonic epilepsy, *TLE* temporal lobe epilepsy.

**Table 3:** Patient characteristics, anamnestic data and the frequency of minor physical anomalies.

	No of patients	Age at epilepsy onset (years)	Gender	Therapy resistance (%)	MPA (total count)	PV (total count)	MM (total count)	ND disorder (epilepsy) in close relatives
Control	30	N/A	14 males (47%)	N/A	M: 1 r: 1–2	M: 0 r: 0–1	M: 0 r: 0–2	0 (0)
Acquired epilepsy	63	M: 38 r: 7–74	30 males (48%)	18 (29%)	M: 1 r: 0–5	M: 0 r: 0–2	M: 1 r: 0–3	2 (2)
TLE with HS	27	M: 22 r: 1–51	11 males (41%)	22 (81%)	M: 2 r: 0–5	M: 1 r: 0–3	M: 1 r: 0–3	1 (1)
Epilepsy with cortical dysgenesis	29	M: 12 r: 1,5–57	13 males (45%)	25 (86%)	M: 3 r: 1–6	M: 2 r: 0–4	M: 1 r: 0–6	1 (0)
Cryptogenic epilepsy	69	M: 19 r: 3–75	29 males (42%)	22 (32%)	M: 2 r: 0–6	M: 1 r: 0–4	M: 1 r: 0–4	2 (2)
**IGE**								
JME	21	M: 14 r: 6–26	3 males (14%)	11 (52%)	M: 4 r: 1–6	M: 2 r: 0–3	M: 2 r: 0–4	6 (3)
CAE	1	1	1 female	0	3	2	1	0 (0)
JAE	12	M: 10 r: 2–19	4 males (33%)	6 (50%)	M: 3 r: 1–4	M: 1 r: 0–2	M: 2 r: 0–3	2 (1)
E-GTCS	13	M: 15 r: 4–37	7 males (54%)	2 (15%)	M: 4 r: 1–6	M: 2 r: 0–3	M: 2 r: 0–4	1 (1)

*CAE* childhood absence epilepsy, *E-GTCS* epilepsy with generalized tonic–clonic seizures, *HS* hippocampal sclerosis, *IGE* idiopathic generalized epilepsy, *JAE* juvenile absence epilepsy, *JME* juvenile myoclonic epilepsy, *M* median, *MM* minor malformations, *MPA* minor physical anomalies, *ND* neurodevelopmental disorder, *No* number, *PV* phenogenetic variants, *r* range, *TLE* temporal lobe epilepsy, *y* year.

### Frequency of MPAs

**Figure 1:**
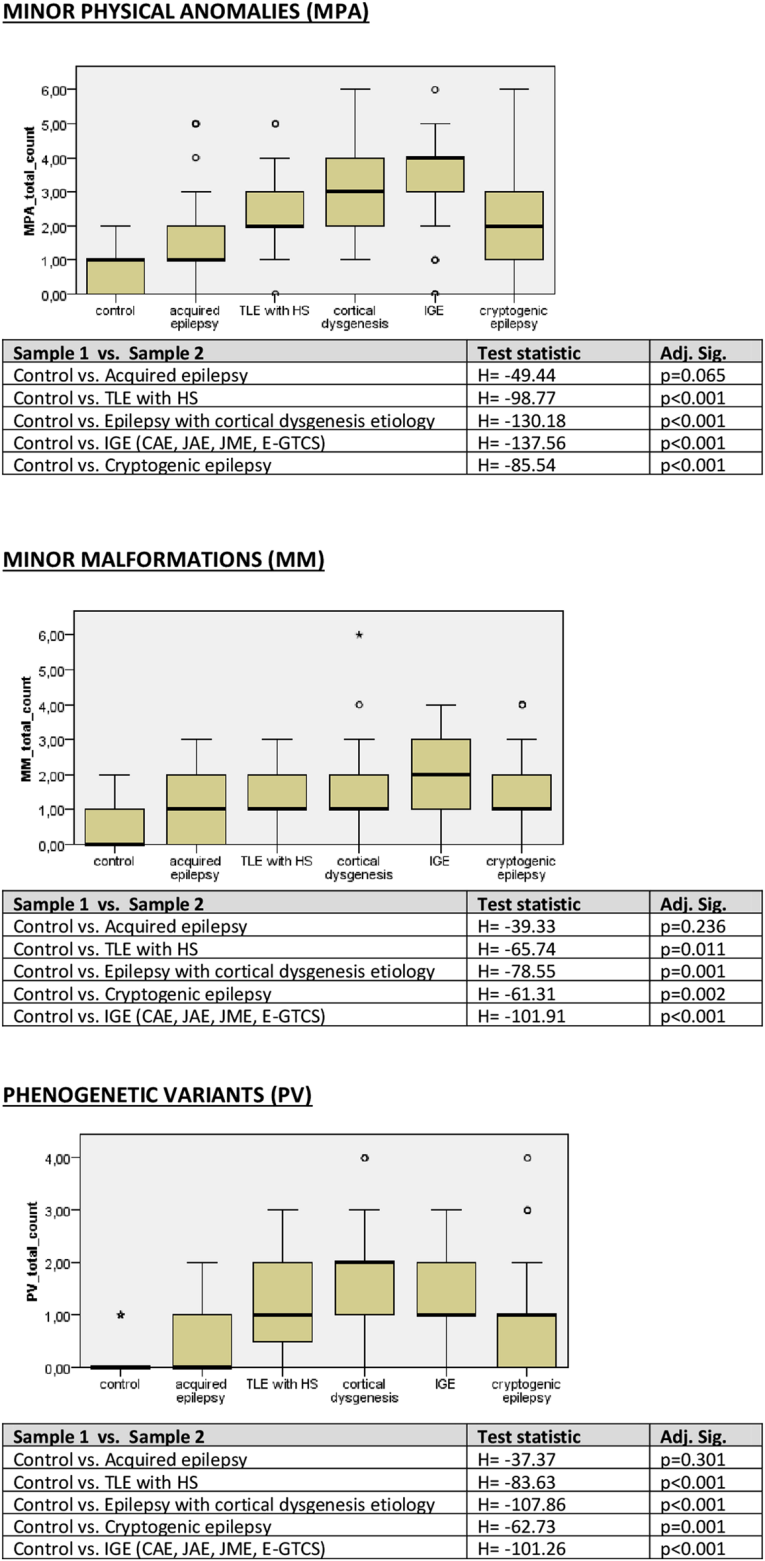
Results of the Kruskal–Wallis test pairwise comparisons in terms of the frequency of MPAs, MMs, as well as PVs between the patient groups compared to control (4 IGE syndromes taken together).

**Figure 2:**
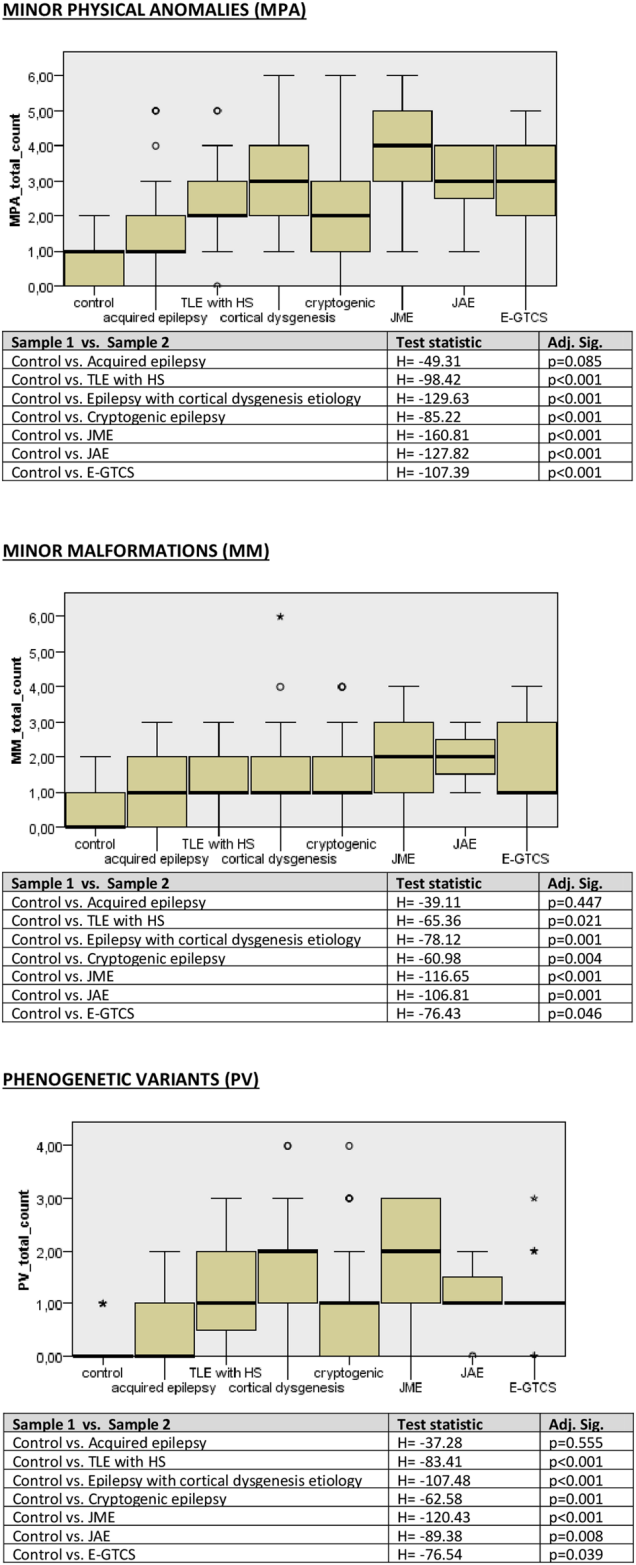
Results of the Kruskal–Wallis test pairwise comparisons in terms of the frequency of MPAs, MMs, as well as PVs between the patient groups compared to control (4 IGE syndromes taken sparately).

The frequency of MPAs (that is the MM and PV altogether), MMs and PVs were significantly affected by the type of epilepsy [H(6) = 90.17; p < 0.001]. Pairwise comparisons with adjusted p values showed that all patient groups except for acquired epilepsy were associated with increased frequency of MPAs, MMs, as well as PVs (Fig. 1). In a following analysis, the IGE subgroups were included in the Kruskal–Wallis test separately (except for CAE as it contained only 1 case), the results are displayed in Fig. 2. To assess the difference of the frequency of MMs and PVs between the epilepsy subgroups (TLE with HS, epilepsy with cortical dysgenesis etiology, cryptogenic epilepsy and IGE), multinomial logistic regression model was designed with the inclusion of age at epilepsy onset, gender and the occurrence of MMs and PVs as independent variables. There proved to be no difference in the occurrence of MMs and PVs among the different types of epilepsies, except for cryptogenic epilepsy and IGE, in which case PVs were more common in the latter [Exp(B) = 3.62; CI 95% 1.26–10.40; p = 0.017].

### Type of MPAs

The occurrence of each MPA was also compared between the epilepsy subgroups and controls: Table 4 displays the results of the two-tailed Fisher exact tests before and after Bonferroni correction, where the adjusted p value was set to 0.001. Furrowed tongue and high arched palate proved to be more common compared to controls even after Bonferroni correction in all epilepsy subgroups except for TLE (Table 4). These two MPAs were further analyzed by comparing their occurrence across the different subgroups of epilepsy. Bonferroni correction was again applied to correct for multiple comparisons (the adjusted p value was set to 0.005). The analysis showed no difference in the occurrence of furrowed tongue and high arched palate among the different epilepsy subgroups (9 Fisher exact tests were performed with a significance level of > 0.007 in each case).

**Table 4:** Occurrence of each minor physical anomaly in each epilepsy subgroup compared to controls. Those signs that remained significant even after Bonferroni correction are highlighted in bold.

**TLE with HS**
Haemangioma (p = 0.021)
Furrowed tongue (p = 0.008)
High arched palate (p = 0.003)
**Epilepsy with cortical dysgenesis**
Cup ears (p = 0.005)
**Furrowed tongue (p < 0.001) !**
**High arched palate (p < 0.001) !**
**Cryptogenic epilepsy**
**Furrowed tongue (p < 0.001) !**
**High arched palate (p = 0.001) !**
**IGE (CAE, JAE, JME, E-GTCS altogether)**
Flat occiput (p = 0.042)
Cup ears (p = 0.038)
Haemangioma (p = 0.042)
Multiple buccal frenula (p = 0.010)
**Furrowed tongue (p < 0.001) !**
**High arched palate (p < 0.001) !**
Sole crease (p = 0.005)
**JME**
Asymmetrical size of ears (p = 0.024)
Cup ears (p = 0.024)
Multiple buccal frenula (p = 0.003)
**Furrowed tongue (p = 0.001) !**
**High arched palate (p < 0.001) !**
Wide distance between the 1st and 2nd toe (p = 0.004)
Sole crease (p = 0.001)
**JAE**
Multiple buccal frenula (p = 0.019)
**Furrowed tongue (p = 0.001) !**
High arched palate (p = 0.004)
**E-GTCS**
Haemangioma (p = 0.024)
**High arched palate (p < 0.001) !**

*CAE* childhood absence epilepsy, *E-GTCS* epilepsy with generalized tonic–clonic seizures, *HS* hippocampal sclerosis, *IGE* idiopathic generalized epilepsy, *JAE* juvenile absence epilepsy, *JME* juvenile myoclonic epilepsy, *TLE* temporal lobe epilepsy.

In case of patients with cortical dysgenesis etiology, no association could be detected between the affected brain lobe and the frequency of MMs and PVs (p = 0.658; p = 0.189, respectively). To assess the relation between the occurrence of MMs and PVs and antiepileptic drug therapy resistance, binary logistic regression model was designed with the inclusion of gender, age and etiology as independent variables: a positive association between the presence of MMs and therapy resistance [Exp(B) = 4.19; CI 95% 1.37–12.80; p = 0.012] was detected. Analyzing the patients’ family history, there proved to be no association between the occurrence of MPAs, MMs and PVs and the presence of neurodevelopmental disorders (see “Methods”) either in direct or collateral relatives [p = 0.51; p = 0.23; p = 0.17, respectively and p = 1; p = 1; p = 0.12, respectively].

## Discussion

To our best knowledge, this is the first study to investigate the rate and topological profile of minor physical anomalies (MPAs) in adult patients with epilepsy with the use of a comprehensive modern scale of dysmorphology. With the application of the Méhes scale we were able to investigate the prevalence of 57 MPAs and to differentiate them as either minor malformations (MMs) or phenogenetic variants (PVs). MMs, arising during organogenesis are qualitative, all-or-none defects that are always regarded as abnormal. In contrast, PVs are quantitative defects of final morphogenesis, developing after organogenesis. They are considered as exact equivalents of normal anthropometric variants, as opposed to MMs.

Few reports are available from the first part of the twentieth century describing increased frequency of MPAs in epilepsy patients^24–26^. Epicanthic fold’s occurrence rate of 43% was found in intellectually normal adults with epilepsy^26^, moreover, by studying offspring of epileptic mothers, unambiguous evidence was obtained about the inheritance of epicanthus, independently of the in utero drug exposure^31^.

We have found a significantly higher number of MPAs in all epilepsy subgroups, except for acquired epilepsy. The overrepresentation of these anomalies supports the theory that epilepsy is related to factors early in development. However, in case of acquired cases the insult seems to impact an intact nervous system indeed, without a possible predisposing effect of a neurodevelopmental abnormality. Since both MMs and PVs have been proven to be significantly more common in each subgroup of epilepsy (except for acquired epilepsy), it indicates that the impact of the given insult is not just limited to a single developmental step but rather owns a long-acting effect and induces complex changes in neurodevelopment. It seems important to underline that the two MPAs that were significantly more common in epilepsy patients (furrowed tongue and high arched palate), both were located in the head region. Furthermore, another 4 out of the 9 MPAs, that showed significant differences compared to controls without Bonferroni correction, also involved the head (cup ears, flat occiput, asymmetric size of ears, multiple buccal frenula). Previous MPA studies pointed out that increased frequency of MPAs of the mouth and the head has a huge relevance to the hypothetical neurodevelopmental failure^10,16^. These current results correspond to those of our previous study on children, in which all three MPAs that were significantly more common in epilepsy patients, were situated in the head region^27^.

Multinomial logistic regression model detected no significant difference in the occurrence of MPAs, MMs and PVs among the different epilepsy subgroups, except between cryptogenic epilepsy and IGE, with PVs being more common in the latter. This result, as well as the robust finding (Table 2) that the MPA count of ≥ 4 was by far the most common in IGE (especially JME) patients, somewhat suggests that the neurodevelopmental factor is the most decisive in this disorder.

Our results correspond to the observations of novel studies on the neurodevelopmental role in various epilepsy syndromes^1–3,32–34^. In various diseases with epilepsy being an accompanying symptom, a connection between the abnormal development of the central nervous system and the surface ectoderm has been described previously, for example the characteristic cutaneous lesions in neurofibromatosis or syndactyly in some cases of bilateral periventricular heterotopia^35,36^. Increased facial asymmetry has also been described in epilepsy patients with unilateral lesions^37^. Moreover, in an interesting study it was found that epilepsy patients with pathogenic genomic structural variants had significantly more atypical face shape than those without, as detected by 3D stereophotogrammetry and dense surface models^38^. Recently it has been recognized that apart from cases on grounds of cortical dysgenesis, abnormal prenatal proliferation, migration and organization of neuronal and glial cells and impairment of postnatal maturation of neuronal networks also contribute to the epileptogenesis, even in epilepsy syndromes that were previously assumed to be caused by one specific anomaly. This implies the complex nature of epileptogenesis, being the result of a combination of multiple genetic and environmental factors—with different rates of these two in each epilepsy syndrome. Grey matter alterations in both cortical and subcortical structures, as well as anomalies in white matter integrity have been described in JME already at the time of seizure onset^2^. This overthrew the simplistic pathophysiological concept of JME as a disrupted balance between excitation and inhibition in an otherwise histologically normal brain and implied that altered neurodevelopment in the embryonic stage also contributes to the pathogenesis. Ventricular enlargement and distinct white and grey matter abnormalities have also been described in CAE, JAE and E-GTCS^32–34^. Extratemporal microdysgeneses in temporal lobe epilepsy with hippocampal sclerosis have also been detected^3^. In patients who underwent epilepsy surgery, an association between microdysgenesis and early age at seizure onset, as well as intellectual disability was described^4^. A study reported significantly higher density of neurons in the white matter of patients with temporal lobe epilepsy than in that of controls and proposed proposed important role of cortical maldevelopment in TLE with HS^39^. The recent discovery of these developmental factors’ additional role in pathogenesis gave rise to the concept of epilepsy as a neurodevelopmental disorder^1^.

Despite the release of new antiepileptic drugs, pharmacoresistance—affecting each epilepsy syndrome with various degree—is still an unresolved issue in epilepsy management. Lately the disease modifying strategy is coming into prominence serving with potential tools to obtain seizure-freedom in patients with pharmacoresistant epilepsy^40,41^. An interesting example is the reestablishment of cortical layering by postnatal re-expression of doublecortin (DCX) gene in subcortical band heterotopia^42^. Thus, the need for thorough exploration of the underlying etiology in different epilepsy syndromes has become a matter of course, especially in the light of the increasing amount of evidence in support of the concept of epilepsy being a neurodevelopmental disorder. The pathomechanism of drug resistance has been mainly studied according to two main hypotheses, namely “drug transporter overexpression” and “reduced drug-target sensitivity”, both implying drug resistance as a condition independent from the underlying etiology^43,44^. However, we found an increased frequency of MPAs in drug resistant epilepsy which rather implies the role of an underlying neurodevelopmental factor. Our results support the “intrinsic severity hypothesis”^43^, according to which neurobiological factors contribute to both epilepsy severity and drug resistance. Treatment resistance was found to correlate with the increased prevalence of MPAs in schizophrenia, too^45^.

## Conclusion

Minor physical anomalies are more common in patients with epilepsy, which corroborates the emerging concept of epilepsy as a neurodevelopmental disorder. Assessment of these signs may contribute to the clarification of the underlying etiology, possibly opening new doors for achieving seizure freedom in the frame of the trending disease modifying approach of disease control.

## Data Availability

The datasets generated and/or analysed during the current study are not publicly available due to the Law of Hungary, according to which raw data of patients cannot be displayed in an open data repository, but are available from the corresponding author on reasonable request.
